# Failure of sucrose replacement with the non-nutritive sweetener erythritol to alter GLP-1 or PYY release or test meal size in lean or obese people

**DOI:** 10.1016/j.appet.2016.09.009

**Published:** 2016-12-01

**Authors:** Joost Overduin, Tinh-Hai Collet, Nenad Medic, Elana Henning, Julia M. Keogh, Faye Forsyth, Cheryl Stephenson, Marja W. Kanning, Rianne M.A.J. Ruijschop, I. Sadaf Farooqi, Agatha A. van der Klaauw

**Affiliations:** a NIZO Food Research, Ede, The Netherlands; b University of Cambridge Metabolic Research Laboratories, Wellcome Trust-Medical Research Council Institute of Metabolic Science, Addenbrooke's Hospital, Cambridge, United Kingdom; c Department of Psychiatry, University of Cambridge, Cambridge, United Kingdom

**Keywords:** Sweeteners, Erythritol, Glucagon-like peptide 1, Peptide YY, Obesity

## Abstract

There is considerable interest in the effect of foods containing high intensity sweeteners on satiation. However, less is known about low-calorie bulk sweeteners such as erythritol. In this randomized three-way crossover study, we studied 10 lean and 10 obese volunteers who consumed three test meals on separate occasions: (a) control sucrose meal; (b) isovolumic meal with partial replacement of sucrose by erythritol; (c) isocaloric meal which contained more erythritol but equivalent calories to the control meal. We measured gut hormone levels, hunger and satiety scores, *ad libitum* food intake, sucrose preference and intake after the manipulations. There was a greater post-prandial excursion in glucose and insulin levels after sucrose than after the erythritol meals. There was no difference in GLP-1/PYY levels or subsequent energy intake and sucrose preference between sucrose control and isovolumic erythritol meals. In lean (but not obese) participants, hunger decreased to a greater extent after the isocaloric erythritol meal compared to the control meal (p = 0.003) reflecting the larger volume of this meal. Replacing sucrose with erythritol leads to comparable hunger and satiety scores, GLP-1 and PYY levels, and subsequent sucrose preference and intake.

## 1 Introduction

The use of low-caloric and non-caloric sweeteners as substitutes for sucrose has increased markedly in the past decades, reflecting widespread attempts to reduce energy consumption and combat the rising prevalence of obesity (Mattes and Popkin, 2009, Sadler and Stowell, 2012). However, several common high-intensity sweeteners (HIS), e.g. aspartame, sucralose and saccharin have been the subjects of enduring controversy due to observational and laboratory studies indicating an association of HIS consumption with increased appetite, food intake, weight gain and glucose intolerance (Bellisle and Drewnowski, 2007, Benton, 2005, Suez et al., 2014, Swithers et al., 2010). In some studies, these paradoxical orexigenic effects of sweeteners occurred after repeated consumption. In other studies, effects of sweeteners occurred after single episodes of consumption (Rogers, Carlyle, Hill, & Blundell, 1988).

In contrast, few studies have addressed these effects after the use of low-calorie bulk sweeteners (LCBS) that are used in solid foods, e.g. desserts, pastries, chocolates, and also occur naturally in fruit and vegetables. LCBS such as xylitol, maltitol, lactitol and erythritol are similarly or less sweet than sucrose, and may be included in foods at several hundred-fold higher doses than HIS. In the food industry, LCBS are used to enhance the physical structure and mouthfeel of foods and to lower the glycaemic index and energy density of sucrose-reduced foods (Livesey, 2003, de Cock, 2012). Erythritol (1,2,3,4-butanetetrol) is a simple polyol that stands out among the LCBS because of its more complete gut absorption and tolerability and renal clearance without any metabolic change which makes it essentially non-caloric (Munro et al., 1998, de Cock, 2012). However, there is little information about whether erythritol has effects on appetite, gut hormone profiles or food preference as is seen with HIS in humans.

We aimed to study the short-term, satiety-related physiological and behavioural effects of erythritol. We performed a randomized three-way crossover study in which lean and obese volunteers were given a test breakfast replacing sucrose with erythritol. The two test meals containing the sweetener were matched for volume or total energy content to the sucrose containing test meal. In addition to perceived hunger and satiety, we investigated the effects of these manipulations on postprandial gut hormone secretion. We were also interested in whether erythritol consumption would alter food intake and preference and therefore measured total energy intake and sucrose preference during a subsequent *ad libitum* meal.

## 2 Material and methods

Ten lean (body mass index, BMI, <25 kg/m^2^) and ten obese (BMI >30 kg/m^2^) volunteers who were weight stable, participated in this randomized three-way crossover study: lean volunteers (5 females and 5 males) had a mean age of 33.4 years (range 26.3–47.0) and a mean BMI of 21.8 kg/m^2^ (SD 2.0); obese volunteers (5 females and 5 males) had a mean age of 34.6 years (range 24.9–46.7) and a mean BMI of 34.2 kg/m^2^ (SD 3.6). Exclusion criteria were the use of any medication, the presence of any medical illnesses including diabetes, lactose intolerance or any food allergies. Written informed consent was obtained prior to the study and ethical approval was obtained from the Local Regional Ethics Committee in Cambridge, UK. The study was conducted in accordance with the principles of the Declaration of Helsinki.

### 2.1 Composition of sucrose and erythritol containing test breakfast

The breakfast test meals were semi-solid custard allowing the manipulation of sucrose/erythritol content and the delivery of the required volume for each volunteer. The test meals were produced by NIZO Food Research B.V. (Ede, The Netherlands) using a production protocol with hazard analysis and critical control points (HACCP) principles. Their macronutrient profile, caloric values and volumes are shown in Table 1. All meals were produced using fresh skimmed milk standardized with cream and skimmed milk powder (all from Friesland Campina, The Netherlands), gelatin (220 Bloom, Gelita Deutschland GmbH, Eberbach, Deutschland), and modified maize starch (C*PolarTex 06741, Cargill, Sas van Gent, The Netherlands) for texture, vanilla flavour (J.B. de Lange, Belfeld, The Netherlands) and colorant (BC-2S-WSS orange, Chr. Hansen, Denmark). The sucrose control meal was sweetened with sucrose (SuikerUnie, Breda, The Netherlands). The erythritol meal was sweetened with erythritol (Zerose™ Erythritol, Cargill, Vilvoorde, Belgium), sucrose and sucralose (Tate & Lyle, United Kingdom).

The sucrose control meal had an energy density of 506.3 kJ/100 g (121 kcal/100 g) and contained 10 g of sucrose per 100 g of meal (10% wt/wt). In erythritol meals, 80% of the sucrose was replaced by erythritol on a weight-for-weight basis. Consequently, the erythritol meals contained 8% (wt/wt) erythritol and 2% (wt/wt) sucrose and had a 25% lower energy density than the sucrose meal. Because the sweetness level of erythritol is not the same as sucrose, some sucralose was added, while the small amount of sucrose counteracted any potential aftertaste of erythritol. The erythritol meal contained about half of the carbohydrates of the sucrose-control meal per unit of weight (Table 1). All meals contained equal amounts of lactose (5% wt/wt) and starch (2% wt/wt) to optimize texture and palatability. Fat and protein contents were also equal in both meals.

Care was taken to match the sweetness of the erythritol and sucrose control meals. Because erythritol provides approximately 0.7 times the sweetness of sucrose (Goossens & Röper, 1994), it is often combined with small doses of HIS. We added 0.004 g sucralose per 100 g of the erythritol food, representing a 13–16 mg dose of sucralose for the entire meal. Human studies with higher doses of sucralose than this have shown no effects on glucagon-like peptide-1 (GLP-1) (Ma et al., 2009, Pepino et al., 2013, Wu et al., 2013) or peptide YY (PYY) secretion (Ford et al., 2011).

### 2.2 Study conduct

In a randomized three-way crossover study design, lean and obese volunteers consumed the following test breakfast on different occasions (a) the sucrose control meal, (b) an isovolumic (reduced calorie) serving of the erythritol meal, (c) an isocaloric (larger volume) serving of the erythritol meal to match for calories to the control meal. The latter condition was added to distinguish sweetener-related effects from those of the reduction in energy content. The three test sessions were allocated by simple randomization and separated by at least one week. Subjects were sequentially allocated and blinded, although the isocaloric test meal implied a larger volume. Subjects were fasted from 22:00 h the night before the study and admitted to the National Institute for Health Research Wellcome Trust Clinical Research Facility in Addenbrooke's Hospital, Cambridge, UK at 07:00 h. This is a controlled clinical research environment where exposure to external food stimuli such as food smell, pictures of food in magazines, and food programmes or commercials on TV are prohibited during the studies. An intravenous indwelling cannula was inserted and volunteers rested for approximately thirty minutes. Blood was drawn and visual analogue scores (VAS) were completed to assess hunger and fullness half-hourly from 07:30 h until 12:00 h. The 7:30 h and 8:00 h measurements were averaged for a baseline measurement before breakfast was given. Test breakfasts were given at 08:00 h and subjects were instructed to finish them within 20 min. Visual analogue scores (VAS) were measured on a 100-mm long line for liking of the meal and sweet and savoury sensations to assess potential within- and between-meal sensory differences after a taster (mouthful of meal) and after completion of the test breakfast. The sucrose control and erythritol test meals were rated as equally palatable after the taster for both groups (F(2,36) = 0.87, Greenhouse-Geisser ε = 0.66, p = 0.39, Fig. S1). The sucrose- and erythritol-containing meals were rated after the taster and after full consumption as equally sweet (F(2,36) = 0.59, ε = 0.68, p = 0.50, Fig. S2) and savoury (F(2,36) = 0.75, ε = 0.79, p = 0.45, Fig. S3).

The calories given for the sucrose control meal were standardized for each participant to match 20% of the individually calculated energy requirements, as calculated by Schofield equations for basal metabolic rate (Schofield, 1985), multiplied by a physical activity index of 1.25. The Schofield equations take into account the age, gender and weight of the individual. An *ad libitum* buffet lunch was served at 12:30 h. The lunch consisted of three common, widely preferred food items (two savoury and one sweet: chicken korma, sweet and sour chicken, orange cake). There were two versions of each item designed to provide low sucrose and high sucrose content; meals were covertly weighed before and after consumption (Table 2). All six food items were presented to participants at the same time and they were instructed to select items at will and eat until comfortably full (in individual rooms). The consumption of each food item was then covertly weighed.

Blood was collected in EDTA tubes containing 100 μL of aprotinin (for PYY and total GLP-1), lithium heparin tubes (insulin) and fluoride oxalate tubes (glucose). Plasma samples were centrifuged immediately at 4 °C and stored at −80 °C until assays were performed. Plasma glucose was assayed on the same day by using the glucose oxidase method. Insulin was quantified using a commercially available immunoassay (AutoDELFIA Insulin Kit; Perkin Elmer, Wellesley, MA), which has an intra-assay coefficient variation of 3.5–4.5%. Plasma PYY and total GLP-1 were measured using an established in-house radioimmunoassay described previously (Adrian et al., 1985, Kreymann et al., 1987). The detection limit of the PYY and total GLP-1 assays was 2.5 and 7.5 pmol/l with an intra-assay coefficient variation of 5.8 and 5.4%, respectively.

### 2.3 Statistical analyses

Data are presented as mean and standard error of the mean (SEM) and were analysed using Stata software package (version 12.1, StataCorp, College Station, TX). We compared the area under the curve (AUC) calculated with trapezoidal integration for the VAS, glucose, insulin and the gut hormones between the three breakfasts. For glucose and insulin, we also compared the peak (maximum) values between the three breakfasts. First, lean and obese participants were analysed separately by analysis of variance (ANOVA) with repeated measures to test for within-subjects changes and between breakfast differences. Subsequently, multivariate ANOVA with repeated measures was performed to investigate group differences between lean and obese volunteers with test meal as within-subjects factor and group as between subjects' factor. The within-subjects p-value was adjusted using the Greenhouse-Geisser correction factor (ε) for lack of sphericity. Pairwise comparisons of the study phases were performed by two-sided Student's t-test when appropriate. A p-value of 0.05 was considered significant after Bonferroni correction for multiple comparisons.

The sample size included 10 lean and 10 obese volunteers based on effect differences commensurate with our own studies and studies in the literature (Batterham et al., 2006, Lomenick et al., 2009, van der Klaauw et al., 2013). In post-hoc power analysis of repeated measures ANOVA in a crossover study with this sample size, the power ranged between 74% and 99.8% for hormone AUC comparisons (except for PYY AUC in obese participants: 33%).

## 3 Results

### 3.1 Hunger and fullness ratings

**Fig. 1:**
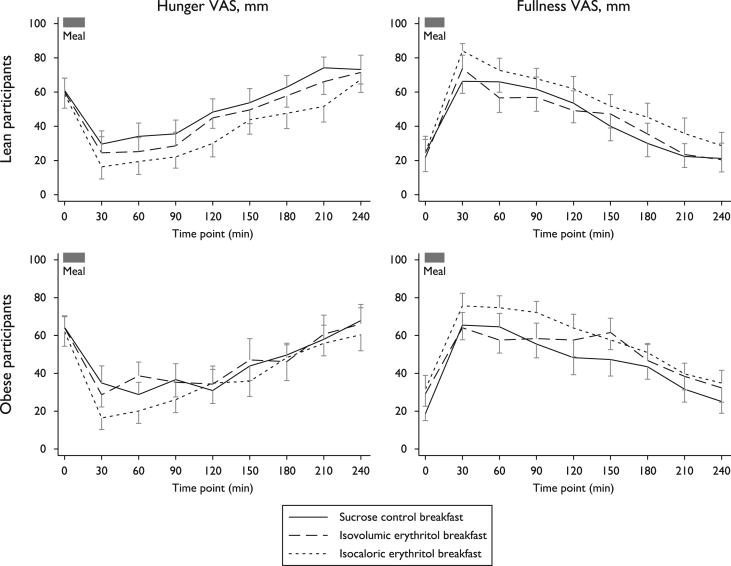
Hunger and fullness scores after test breakfast, by type and group. Data are presented as mean ± standard error of the mean. In lean participants, hunger (top-left panel) and fullness scores (top-right panel) based on visual analogue scores (VAS) half-hourly after test breakfast consumption did not differ between the sucrose control (solid line) and the isovolumic erythritol meal (dashed line), while hunger scores were lowest and fullness scores highest after the isocaloric erythritol meal (dotted line, p = 0.003 and p = 0.02, respectively). In obese subjects, hunger and fullness scores were similar after consumption of the different test meals (bottom panels).

In lean participants, AUC of hunger and fullness scores did not differ between the sucrose control and the isovolumic erythritol meal (Fig. 1). However, hunger scores were lowest and fullness scores were highest after the isocaloric erythritol meal (F(2,18) = 7.78, ε = 0.99, contrast p = 0.003 for hunger; F(2,18) = 6.61, ε = 0.89, contrast p = 0.02 for fullness) reflecting the larger volume of this meal. In contrast, AUC of hunger and fullness scores were similar after consumption of the different meals in obese participants (F(2,18) = 1.08, ε = 0.99, p = 0.36 for hunger; F(2,18) = 2.61, ε = 0.89, p = 0.11 for fullness, Fig. 1). No side effects were observed after the erythritol meals.

### 3.2 Glucose and gut hormone response

**Fig. 2:**
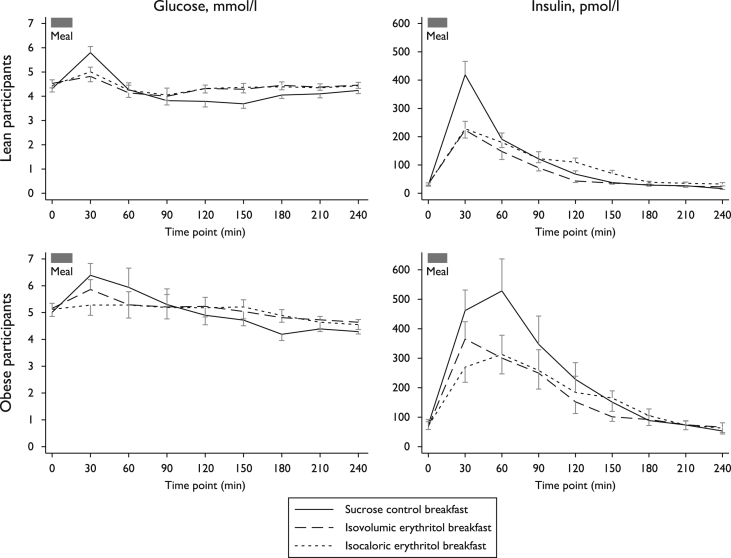
Glucose excursion and insulin secretion after test breakfast, by type and group. Data are presented as mean ± standard error of the mean. Plasma glucose excursion (left panels) and insulin secretion (right panels) peaked the most after sucrose control meal (solid line) compared to the sweetener meals (dashed and dotted lines, all p ≤ 0.007) in both study groups.

The glucose peak concentration was highest after the sucrose control meal (mean 5.81, SEM 0.25 mmol/l in lean subjects; mean 6.66, SEM 0.55 mmol/l in obese subjects) compared to the sweetener meals (mean 5.03, SEM 0.11 mmol/l, F(2,18) = 9.57, ε = 1.00, all contrast p ≤ 0.006 in lean; mean 5.88, SEM 0.27 mmol/l, F(2,18) = 12.91, ε = 0.75, all contrast p ≤ 0.007 in obese participants; Fig. 2). Starting from similar levels of pre-meal glucose (all p ≥ 0.18), 2 h postprandial glucose was significantly lower than pre-meal baseline after the sucrose meal in lean subjects (mean 3.79, SEM 0.23 mmol/l) but not after erythritol meals (mean 4.32, SEM 0.16 mmol/l, F(2,18) = 9.91, ε = 1.00, all contrast p < 0.003). In keeping with these observations, insulin secretion was highest after the sucrose control compared to the erythritol-containing meals (F(2,36) = 25.78, ε = 0.84, all contrast p ≤ 0.005 for peak levels; F(2,36) = 9.62, ε = 0.76, all contrast p < 0.04 for insulin AUC).

**Fig. 3:**
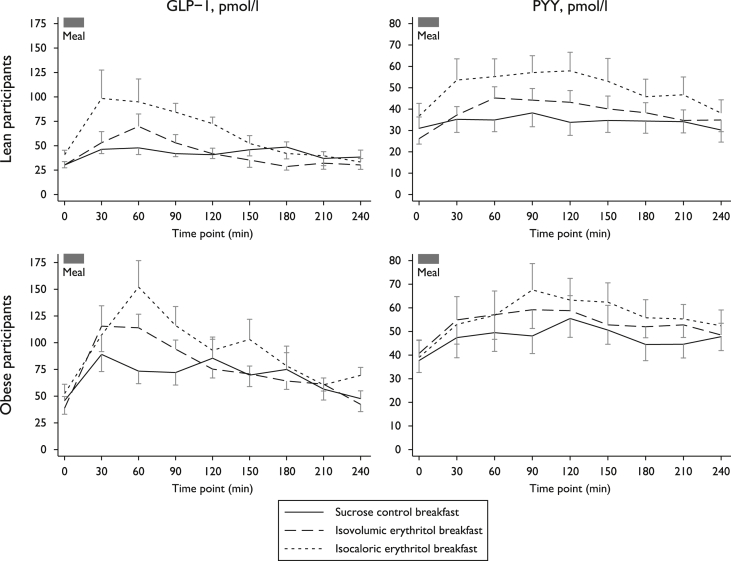
PYY and GLP-1 secretion after test breakfast, by type and group. Data are presented as mean ± standard error of the mean. In lean subjects, gut hormones glucagon-like peptide-1 (GLP-1, top-left panel) and peptide YY (PYY, top-right panel) increased similarly after the isovolumic erythritol (dashed line) and the sucrose control test breakfasts (solid line, p ≥ 0.76), while they increased more markedly after isocaloric erythritol meal consumption (dotted line, all p ≤ 0.04). The satiety response of GLP-1 and PYY after the different meals was similar in obese participants (bottom panels).

In lean and obese subjects, the baseline levels of gut hormones GLP-1 and PYY were similar (all p ≥ 0.10) and postprandial increases of AUC were similar for the isovolumic erythritol and the sucrose control meals (contrast p ≥ 0.47, Fig. 3 and Table 3 with F statistics). The isocaloric (larger volume) erythritol meal was associated with a more marked postprandial increase in AUC of both hormones in lean subjects (contrast p ≤ 0.04 for GLP-1; contrast p ≤ 0.04 for PYY), but not in obese participants (all p ≥ 0.10 for GLP-1 and PYY). GLP-1 peak levels did not differ between test meals (p = 0.15 in lean and p = 0.18 in obese subjects), while they differed for PYY peak levels after the isocaloric sweetener meal (contrast p ≤ 0.02 in lean and p = 0.04 in obese subjects).

In multivariate repeated measures ANOVA comparing the lean and obese groups, we found higher AUCs of plasma glucose (F(1,18) = 6.33, p = 0.02), insulin (F(1,18) = 9.04, p = 0.008), and GLP-1 secretion (F(1,18) = 15.37, p = 0.001) in the obese participants. Hunger and fullness scores and PYY secretion were not different between the two groups.

### 3.3 Food intake and sucrose preference at subsequent meal

To test if replacing sucrose by erythritol affected subsequent preference for sucrose, we provided subjects with an *ad libitum* food buffet four hours after consumption of the breakfasts. A low vs. high sugar version of three popular food items was provided in excess and the consumption was covertly weighed. Two lean volunteers were not included in this analysis because of doubts about the exact amount of food consumed. Energy intake during *ad libitum* lunch buffet intake did not differ between the three test breakfasts in lean and obese participants (Table 2; F(2,14) = 0.52, ε = 0.90, p = 0.59; and F(2,18) = 0.42, ε = 0.87, p = 0.64 respectively). Also, no effect was found on preference for low versus high sucrose containing foods (Table 2, all p ≥ 0.07). This indicates that the erythritol-containing preload meals did not provoke a compensatory increase in energy intake or increased sucrose preference at a subsequent meal in this paradigm.

## 4 Discussion

To our knowledge, this is one of the first studies investigating the effects of partial replacement of sucrose by the non-nutritive bulk sweetener erythritol in humans. We found that the erythritol meals induced a smaller blood glucose excursion and plasma insulin response than the sucrose control meals in our study in line with previous studies of erythritol or other LCBS in mixed meals (Livesey, 2003). However, no differences were found between sucrose control and isovolumic erythritol meals in terms of GLP-1 or PYY release, acute satiation, postprandial hunger and fullness dynamics during the four-hour postprandial period, or subsequent energy intake and sucrose preference during an *ad libitum* lunch buffet. Thus we did not find any evidence that partial replacement of sucrose by erythritol weakened satiety responses or stimulated subsequent food intake or sucrose preference. These findings warrant investigation of the effects of repeated consumption of erythritol on chronic food intake and food preference.

The composition of sucrose control and erythritol meals only differed by the partial (80%) replacement of caloric sucrose by non-caloric erythritol. The similar responses of GLP-1 and PYY to sucrose control and isovolumic erythritol meals suggest that the contained erythritol and sucrose triggered gastrointestinal peptide release equally. This highlights a notable difference between erythritol and HIS which were shown to have no effect on gut hormones GLP-1 and PYY (Ma et al., 2009, Steinert et al., 2011), except for some conflicting results which may be due to methodological differences (Brown & Rother, 2012).

Erythritol potentially activates intestinal sweet-taste receptors like other sweeteners (Jang et al., 2007), but it is likely that this mechanism alone does not fully explain GLP-1 and PYY release after mixed-macronutrient meals. Indeed, the observed gut hormone responses to an erythritol meal suggests that erythritol induces GLP-1 release when it synergizes with the intestinal effects of other co-ingested ingredients as has been shown for HIS (Brown, Walter, & Rother, 2009). Also pointing to this effect is the higher AUC of GLP-1 and PYY levels after the isocaloric, larger volume erythritol meal. This most likely reflects the presence of nutrients (i.e. more carbohydrates, protein and fat) and erythritol that induce hormone release. The absence of this effect in the obese participants is notable and in line with other studies showing alterations in postprandial GLP-1 and PYY secretion in obesity (le Roux et al., 2006, Ranganath et al., 1996).

In addition, our study employed semi-solid foods, which together with solid foods contribute around 30% of the dietary sucrose intake in modern societies (Hu, 2013). Solid foods may have different effects than soda beverages or simple solutions of sweeteners in water, which tend to trigger less consistent hormonal and behavioural satiation responses than solid foods (Peracchi et al., 2000). Further research is needed to elucidate how erythritol may interact with other nutrients to modulate gut hormone secretion.

Several studies have examined the potential effect of sweeteners on subsequent meal consumption with contradictory results, in part explained by varying methodology (Bellisle & Drewnowski, 2007). In our study, the comparable energy intake and sucrose preference observed during an *ad libitum* buffet meal consumed four hours after consumption of the sucrose control and the erythritol meals is notable especially because the isovolumic erythritol meal contained 25% fewer calories. Previously, consumption of aspartame-sweetened soft drinks was found to increase energy intake the following day compared to soft drinks containing sucrose and water, while the appetite ratings were not changed (Lavin, French, & Read, 1997). Pre-load beverages with aspartame or saccharin also greatly increased preferences for savoury and high-protein food compared to plain water (Rogers et al., 1988). To our knowledge, only one recent study tested the effects of LCBS (Wölnerhanssen et al., 2016).

Our study has some limitations. Potential changes in the rate of gastric emptying were not assessed and may partially explain the higher GLP-1 and PYY levels after the isocaloric, larger volume erythritol meal. Due to the limited number of volunteers, the study assessed the AUC of GLP-1 and PYY levels; transient changes in blood levels may have been missed. Notably, comparison of peak gut hormone levels did not reveal major differences. Similarly, the study had limited power to assess small changes in VAS scores. Differences in gastric emptying between lean and obese subjects have been suggested in some but not all studies (Cardoso-Junior et al., 2007); notably, the test breakfasts in our study were semi-solid (custard) which may mean that our data are less likely to be confounded by gastric emptying than would be the case if we had used solid foods. The test breakfasts contained a low amount of sucrose and sucralose in addition to erythritol to match the sweetness of the control breakfast; future studies are needed to test the effects of an erythritol-pure meal.

Overall, these results illustrate the usefulness of evaluating individual sweeteners, which may interact in unique ways with sweet-taste receptors and have different absorption kinetics and metabolic fates after consumption.

## Conflict of interest

Three authors (J.O., M.W.K. and R.M.A.J.R.) were employed by NIZO food research, an independent contract-research organization that carries out research projects for a wide range of dairy, food & beverage, and ingredients companies. No other authors declare a potential conflict of interest.

## Authors' contributions

J.O., A.A.v.d.K., I.S.F. designed the study; M.W.K. helped in the design of the test breakfasts and coordinated the food production; A.A.v.d.K., I.S.F., T.H.C., N.M., E.H., J.M.K., F.F., C.S. conducted the research; A.A.v.d.K., I.S.F., T.H.C. analysed data and performed statistical analyses; J.O., A.A.v.d.K., T.H.C., I.S.F. wrote the paper; all authors contributed to and approved the paper.

## Figures and Tables

**Table 1:** Composition of the test breakfasts.

	Sucrose control breakfast	Isovolumic erythritol breakfast	Isocaloric erythritol breakfast
**Macronutrient composition**			
Energy, kJ/100 g	506.3	372.4	372.4
Protein, g/100 g	4.5	4.5	4.5
Fat, g/100 g	3.9	3.9	3.9
Carbohydrates, g/100 g	17	9	9
Sucrose	10	2	2
Erythritol	–	8	8
Sucralose	–	0.004	0.004
**Mean energy provided (SEM), kJ**			
Lean participants	1643 (90)	1209 (66)	1643 (90)
Obese participants	1999 (95)	1470 (70)	1999 (95)
**Mean breakfast volume (SEM), ml**			
Lean participants	325 (18)	325 (18)	441 (24)
Obese participants	395 (19)	395 (19)	537 (26)

*Footnotes*: In the sucrose replacement breakfasts, the original 10 g of sucrose per 100 g of meal (10% wt/wt) was replaced by 8% (wt/wt) erythritol and 2% (wt/wt) sucrose. A small dose of sucralose (0.004 mg/100 g) was added to match sweetness level of the control sucrose, providing approximately 16 mg per meal. All meals contained equal amounts of lactose (5% wt/wt) and starch (2% wt/wt) to optimize texture and palatability. To convert kiloJoules (kJ) to kcal, divide by 4.184.

**Table 2:** Composition and intake of the *ad libitum* test lunch.

Sucrose content	Chicken korma		Sweet and sour chicken		Orange cake	
	Low (2%)	High (31%)	Low (8%)	High (31%)	Low (16%)	High (32%)
**Energy by macronutrients in %**^a^						
Protein	17.9	12.8	16.1	13.6	12.9	11.0
Fat	44.2	31.4	33.9	33.4	37.1	36.8
Carbohydrates	37.8	55.8	47.9	51.4	49.9	52.1
of which sugars	17.3	60.1	20.0	60.0	33.9	61.9
of which sucrose	1.5	31.4	7.5	30.6	16.3	32.2
**Consumed weight**^b^**(SEM), g**						
Lean participants	209.8 (24.2)	166.7 (20.2)	147.6 (21.4)	135.2 (18.7)	24.0 (7.2)	34.7 (8.2)
Obese participants	210.1 (19.5)	240.7 (33.7)	146.9 (21.5)	150.6 (24.6)	21.7 (5.4)	24.7 (4.5)
**Consumed energy**^b^**(SEM), kJ**						
Lean participants	1448 (167)	1388 (168)	1192 (173)	1104 (153)	271 (81)	402 (95)
Obese participants	1452 (205)	2004 (281)	1186 (174)	1228 (200)	245 (61)	286 (52.3)

a Percentages are energy of single macronutrient as percentage of total energy.

b Data reported in this table are from 8 lean and 10 obese volunteers. To test if replacing sucrose by erythritol affected subsequent preference for sucrose, subjects were provided with an *ad libitum* meal 4 h after consumption of the test breakfasts. The lunch buffet consisted of three items (two savoury, one sweet); there were two versions of each item designed to provide low sucrose and high sucrose content. Two lean volunteers were not included in the analysis of *ad libitum* lunch consumption because of doubts about the exact amount of food consumed. To convert kiloJoules (kJ) to kcal, divide by 4.184.

**Table 3:** Glucagon-like peptide 1 and Peptide YY course after test breakfasts.

Mean (SEM)	Sucrose control breakfast (A)	Isovolumic erythritol breakfast (B)	Isocaloric erythritol breakfast (C)	P values for overall comparison and pairwise contrasts			
				Overall comparison	A vs C	A vs B	B vs C
**Glucagon-like peptide 1**							
Peak level, pmol/l							
Lean participants	82.2 (19.4)	77.6 (11.9)	114.5 (18.2)	F(2,18) = 2.31, ε = 0.69 p = 0.15			
Obese participants	122 (21)	140 (16)	172 (21)	F(2,18) = 1.92, ε = 0.90 p = 0.18			
Area under the curve, pmol/l x mins							
Lean participants	10,903 (1273)	10,319 (1247)	15,263 (1898)	F(2,18) = 5.92, ε = 0.74 p = 0.02	0.04	1.00	0.02
Obese participants	17,045 (2314)	19,065 (1577)	23,137 (2470)	F(2,18) = 2.74, ε = 0.78 p = 0.11			
**Peptide YY**							
Peak level, pmol/l							
Lean participants	44.4 (6.3)	48.7 (5.5)	75.8 (11.5)	F(2,18) = 7.61, ε = 0.64 p = 0.02	0.006	1.00	0.02
Obese participants	61.1 (7.8)	71.0 (9.4)	76.3 (10.2)	F(2,18) = 3.81, ε = 0.96 p = 0.04	0.04	0.28	1.00
Area under the curve, pmol/l x mins							
Lean participants	8199 (1306)	9403 (1134)	12,202 (1712)	F(2,18) = 8.01, ε = 0.99 p = 0.003	0.003	0.77	0.04
Obese participants	11,489 (1580)	12,966 (1615)	13,797 (1901)	F(2,18) = 2.72, ε = 0.98 p = 0.09			

*Footnotes*: Related to Fig. 2. Multivariate ANOVA with repeated measures was performed to investigate group differences between lean and obese volunteers with test meal as within-subjects factor and group as between subjects' factor. The within-subjects p-value was adjusted using the Greenhouse-Geisser (ε) correction factor for lack of sphericity. Pairwise comparisons of the study phases were performed by two-sided Student's t-test when appropriate. A p-value of 0.05 was considered significant after Bonferroni correction for multiple comparisons.
