# Supplementary material for: Failure of sucrose replacement with the non-nutritive sweetener erythritol to alter GLP-1 or PYY release or test meal size in lean or obese people
Source: Appetite. 2016 Dec 1;107:596–603. doi: 10.1016/j.appet.2016.09.009 (PMC5119236; doi:10.1016/j.appet.2016.09.009)
Supplement: Supplementary file 1 [file mmc1.docx]

# Supplementary Information

# Title: Failure of sucrose replacement with the non-nutritive sweetener erythritol to alter GLP-1 or PYY release or test meal size in lean or obese people

### Figure S1. Palatability of the test breakfasts, by type and group

Participants rated the question “How much do you like the pudding?” on a visual analogue score after a taster and full consumption of the test breakfasts. The sucrose control (left panels) and erythritol test meals (middle and right panels) were rated as equally palatable after the taster in both study groups (p=0.39). Data are presented as mean ± standard error of the mean.

### Figure S2. Sweet sensation of the test breakfasts, by type and group

Participants rated the question “How sweet do you find the pudding?” on a visual analogue score after a taster and full consumption of the test breakfasts. The sucrose control (left panels) and erythritol-containing meals (middle and right panels) were rated equally sweet both after the taster and full consumption in both study groups (p=0.68 and p=0.50, respectively). Data are presented as mean ± standard error of the mean.

### Figure S3. Savoury sensation of the test breakfasts, by type and group

Participants rated the question “How savoury do you find the pudding?” on a visual analogue score after a taster and full consumption of the test breakfasts. The sucrose control (left panels) and erythritol-containing meals (middle and right panels) were rated equally savoury both after the taster and full consumption in both study groups (p=0.59 and p=0.42, respectively). Data are presented as mean ± standard error of the mean.
